# Supplementary material for: Markers of protein-energy wasting and physical performance in haemodialysis patients: A cross-sectional study
Source: PLoS One. 2020 Jul 30;15(7):e0236816. doi: 10.1371/journal.pone.0236816 (PMC7392314; doi:10.1371/journal.pone.0236816)
Supplement: S2 Table — (DOCX) [file pone.0236816.s002.docx]

**S2 Table. Patient characteristics according to diabetes.**

| **Variable** | **Patients with diabetes (n=52)** | **Patients without diabetes (n=61)** | ***p*** |
| --- | --- | --- | --- |
|  |  |  |  |
| Age (years) | 71 ± 14 | 63 ± 17 | <0.01 |
| BMI (kg/m^2^) | 28 ± 4 | 24 ± 5 | <0.01 |
| CRP (mg/L) | 8 ± 7 | 5 ± 6 | 0.01 |
| TIBC (µg/dL) | 236 ± 77 | 243 ± 76 | 0.65 |
| Total protein (g/L) | 65 ± 6 | 64 ± 6 | 0.48 |
| Dialysis vintage (months) | 35 ± 33 | 36 ± 40 | 0.86 |
| Number of prescribed medications (n) | 14 ± 3 | 13 ± 4 | 0.53 |
| Davies comorbidity score (0-7) | 2.5 ± 1.1 | 1.5 ± 1.4 | <0.01 |
| Quadriceps force (N) | 159 ± 62 | 182 ± 95 | 0.13 |
| Quadriceps force (%) | 51 ± 19 | 52 ± 22 | 0.66 |
| Handgrip force (kg) | 27 ± 10 | 30 ± 12 | 0.60 |
| Handgrip force (%) | 90 ± 28 | 95 ± 33 | 0.38 |
| DFRI (/12) | 6 ± 3 | 5 ± 3 | 0.08 |
| Tinetti (/12) | 8 ± 4.2 | 8 ± 4.7 | 0.66 |
| FICSIT (/28) | 13 ± 7 | 15 ± 10 | 0.38 |
| Sit-to-Stand (s) | 32 ± 17 | 27 ± 18 | 0.12 |
| 6MWT (m) | 205 ± 164 | 272 ± 209 | 0.07 |
| 6MWT (%) | 36 ± 28 | 43 ± 31 | 0.20 |

Data are reported as mean ± standard deviation; p-values from ANOVA were reported for normal distributed parameters, otherwise they were reported from the Kruskal-Wallis test. Abbreviations: 6MWT, six-minute walking test; BMI, body mass index; DFRI, dialysis fall risk index; CRP, C-reactive protein; TIBC, total iron binding capacity
