# Supplementary material for: Markers of protein-energy wasting and physical performance in haemodialysis patients: A cross-sectional study
Source: PLoS One. 2020 Jul 30;15(7):e0236816. doi: 10.1371/journal.pone.0236816 (PMC7392314; doi:10.1371/journal.pone.0236816)
Supplement: S3 Table — (DOCX) [file pone.0236816.s003.docx]

**Table S3: Detailed association between relevant associations of nutritional measures and domains of physical performance.**

| **Variable** | **Tinetti** | | **STS** | | **6MWT** | |
| --- | --- | --- | --- | --- | --- | --- |
|  | **Estimate (SE)** | ***p* value** | **Estimate (SE)** | ***p* value** | **Estimate (SE)** | ***p* value** |
| MNA | **8.51 (2.36)** | **0.020** | **-40.19 (-2.92)** | **0.004** | **287.30 (2.11)** | **0.038** |
| Total protein | **-11.17 (-3.19)** | **0.002** | 25.77 (1.91) | 0.059 | -76.68 (0.56) | 0.574 |
| TIBC | 0.14 (0.04) | 0.967 | 7.67 (0.57) | 0.567 | -73.46 (-0.54) | 0.589 |
| CRP | -4.03 (-1.14) | 0.256 | 13.07 (0.97) | 0.336 | **-299.21 (-2.20)** | **0.029** |
| BMI | -4.35 (-1.20) | 0.234 | 9.42 (0.68) | 0.497 | -166.97 (-1.22) | 0.224 |
| Data are presented as estimated beta-values and estimated standard error (SE).  *Abbreviations*: 6MWT, six-minute walking test; BMI, body mass index; CRP, C-reactive protein; MNA, mini-nutritional assessment scale; STS, sit-to-stand; TIBC, total iron binding capacity | | | | | | |
