# Supplementary material for: Markers of protein-energy wasting and physical performance in haemodialysis patients: A cross-sectional study
Source: PLoS One. 2020 Jul 30;15(7):e0236816. doi: 10.1371/journal.pone.0236816 (PMC7392314; doi:10.1371/journal.pone.0236816)
Supplement: S4 Table — (DOCX) [file pone.0236816.s004.docx]

**Table S4: Detailed association of relevant associations controlled for age and gender**

| **Variable** | **Tinetti** | | **STS** | | **6MWT** | |
| --- | --- | --- | --- | --- | --- | --- |
|  | **Estimate (SE)** | ***p* value** | **Estimate (SE)** | ***p* value** | **Estimate (SE)** | ***p* value** |
| MNA | **10.60 (2.88)** | **0.005** | **-53.24 (-3.61)** | **< 0.001** | **471.30 (2.98)** | **0.004** |
| Total protein | **-10.91 (-3.15)** | **0.002** | 25.88 (1.89) | 0.062 | -41.51 (0.29) | 0.776 |
| TIBC | 0.73 (0.21) | 0.831 | 5.03 (0.38) | 0.708 | -52.40 (-0.37) | 0.713 |
| CRP | -2.52 (-0.71) | 0.479 | 6.85 (0.49) | 0.627 | -262.61 (-1.75) | 0.083 |
| BMI | -3.72 (-1.02) | 0.311 | 6.64 (0.45) | 0.651 | -149.44 (-0.95) | 0.342 |
| Age | **-12.35 (-3.54)** | **< 0.001** | **63.11 (4.57)** | **< 0.001** | **-849.48 (-5.80)** | **< 0.001** |
| Gender | 5.59 (1.64) | 0.105 | **-40.86 (-3.04)** | **0.003** | **505.37 (3.54)** | **< 0.001** |
| Data are presented as estimated beta-values and estimated standard error (SE), the analyses are controlled for age and gender (male = 1). *Abbreviations*: 6MWT, six-minute walking test; BMI, body mass index; CRP, C-reactive protein; MNA, mini-nutritional assessment scale; STS, sit-to-stand; TIBC, total iron binding capacity | | | | | | |
