# Supplementary material for: Markers of protein-energy wasting and physical performance in haemodialysis patients: A cross-sectional study
Source: PLoS One. 2020 Jul 30;15(7):e0236816. doi: 10.1371/journal.pone.0236816 (PMC7392314; doi:10.1371/journal.pone.0236816)
Supplement: S5 Table — (DOCX) [file pone.0236816.s005.docx]

**Table S5: Detailed association of relevant associations controlled for age, gender and the Davies comorbidities score**

| **Variable** | **Tinetti** | | **STS** | | **6MWT** | |
| --- | --- | --- | --- | --- | --- | --- |
|  | **Estimate (SE)** | ***p* value** | **Estimate (SE)** | ***p* value** | **Estimate (SE)** | ***p* value** |
| MNA | **10.29 (2.91)** | **0.005** | **-53.01 (-3.72)** | **<0.001** | **468.92 (3.10)** | **0.003** |
| Total protein | **-10.60 (-3.15)** | **0.002** | 25.39 (1.91) | 0.059 | -39.22 (0.28) | 0.780 |
| TIBC | -0.20 (-0.06) | 0.953 | 10.29 (0.78) | 0.436 | -11.18 (-0.83) | 0.407 |
| CRP | -1.44 (-0.41) | 0.681 | 0.05 (0.00) | 0.997 | -178.74 (-1.22) | 0.226 |
| BMI | -3.21 (-0.91) | 0.365 | 4.40 (0.31) | 0.757 | -122.72 (-0.82) | 0.416 |
| Age | **-10.47 (-3.02)** | **0.003** | **52.84 (3.83)** | **< 0.001** | **-723.09 (-4.96)** | **< 0.001** |
| Gender | 5.88 (1.77) | 0.080 | **-42.74 (-3.27)** | **0.002** | **526.34 (3.82)** | **< 0.001** |
| Davies score | -5.74 (-1.63) | 0.105 | **33.36 (2.38)** | **0.019** | **-395.82 (-2.67)** | **0.009** |
| Data are presented as estimated beta-values and estimated standard error (SE), the analyses are controlled for age, gender (male = 1) and comorbidities.  *Abbreviations*: 6MWT, six-minute walking test; BMI, body mass index; CRP, C-reactive protein; MNA, mini-nutritional assessment scale; STS, sit-to-stand; TIBC, total iron binding capacity | | | | | | |
