# Supplementary material for: Markers of protein-energy wasting and physical performance in haemodialysis patients: A cross-sectional study
Source: PLoS One. 2020 Jul 30;15(7):e0236816. doi: 10.1371/journal.pone.0236816 (PMC7392314; doi:10.1371/journal.pone.0236816)
Supplement: S6 Table — (DOCX) [file pone.0236816.s006.docx]

**Table S6. Detailed association between measures of nutritional status and physical performance as expected for the patients’ age and gender**

| **Variable** | **Quadriceps strength (%)** | | **Handgrip strength (%)** | | **6MWT (%)** | |
| --- | --- | --- | --- | --- | --- | --- |
|  | **Estimate (SE)** | ***p* value** | **Estimate (SE)** | ***p* value** | **Estimate (SE)** | ***p* value** |
| MNA | **42.43 (2.49)** | **0.014** | 9.53 (0.64) | 0.522 | **72.23 (3.18)** | **0.002** |
| Total protein | 20.91 (1.27) | 0.208 | 3.02 (0.20) | 0.844 | -13.24 (-0.59) | 0.555 |
| TIBC | -9.16 (-0.56) | 0.575 | 1.24 (0.08) | 0.936 | -13.42 (-0.61) | 0.546 |
| CRP | 6.41 (0.39) | 0.700 | -13.83 (-0.91) | 0.364 | **-45.30 (-2.02)** | **0.046** |
| BMI | **-48.03 (-2.80)** | **0.006** | 23.75 (1.61) | 0.111 | -10.34 (-0.45) | 0.652 |
| Data are presented as estimated beta-values and estimated standard error (SE).  *Abbreviations*: 6MWT, six-minute walking test; BMI, body mass index; CRP, C-reactive protein; MNA, mini-nutritional assessment scale; TIBC, total iron binding capacity | | | | | | |
