# Supplementary material for: Markers of protein-energy wasting and physical performance in haemodialysis patients: A cross-sectional study
Source: PLoS One. 2020 Jul 30;15(7):e0236816. doi: 10.1371/journal.pone.0236816 (PMC7392314; doi:10.1371/journal.pone.0236816)
Supplement: S7 Table — (DOCX) [file pone.0236816.s007.docx]

**Table S7. Detailed association controlled for the Davies comorbidities score**

| **Variable** | **Quadriceps strength (%)** | | **Handgrip strength (%)** | | **6MWT (%)** | |
| --- | --- | --- | --- | --- | --- | --- |
|  | **Estimate (SE)** | ***p* value** | **Estimate (SE)** | ***p* value** | **Estimate (SE)** | ***p* value** |
| MNA | **42.61 (2.55)** | **0.012** | 9.60 (0.71) | 0.480 | **83.23 (3.48)** | **< 0.001** |
| Total protein | 21.10 (1.30) | 0.197 | 3.05 (0.22) | 0.828 | -13.07 (-0.57) | 0.573 |
| TIBC | -11.28 (-0.70) | 0.486 | 0.52 (0.04) | 0.970 | -23.99 (-1.04) | 0.298 |
| CRP | 9.89 (0.60) | 0.552 | -11.11 (-0.81) | 0.419 | -30.46 (-1.29) | 0.201 |
| BMI | **-45.81 (-2.72)** | **0.008** | 22.11 (1.64) | 0.103 | -8.01 (-0.33) | 0.740 |
| Davies score | -15.47 (-0.94) | 0.351 | -8.54 (0.62) | 0.538 | **-67.45 (-2.87)** | **0.005** |
| Data are presented as estimated beta-values and estimated standard error (SE), the analyses are controlled for the Davies comorbidities score.  *Abbreviations*: 6MWT, six-minute walking test; BMI, body mass index; CRP, C-reactive protein; MNA, mini-nutritional assessment scale; TIBC, total iron binding capacity | | | | | | |
