# Supplementary material for: Markers of protein-energy wasting and physical performance in haemodialysis patients: A cross-sectional study
Source: PLoS One. 2020 Jul 30;15(7):e0236816. doi: 10.1371/journal.pone.0236816 (PMC7392314; doi:10.1371/journal.pone.0236816)
Supplement: Attachment — Submitted filename: Response To Reviewers.docx [file pone.0236816.s010.docx]

# REVIEWER #1 and #3

We thank the reviewers for their feedback.

# REVIEWER #2

**1) I would suggest that you change the title of the manuscript to: Markers of Protein-Energy Wasting and Physical Performance in Haemodialysis Patients: A Cross-Sectional Study. This is more accurate than using "Nutritional Status" in the title since strictly speaking you are not measuring nutritional status.**

We agree with the reviewer. Changing “nutritional status” to “markers of protein-energy wasting” is more in line with the true message of our manuscript.

The following adjustments were made in the title of the revised version of the manuscript:

Title page, page 1, lines 1-2: “Markers of protein-energy wasting and physical performance in haemodialysis patients: a cross-sectional study”
